# The Lesions Associated with Dysmenorrhea

**Published:** 1895-12

**Authors:** William S. Gardner

**Affiliations:** Baltimore, Md.


### THE LESIONS ASSOCIATED WITH DYSMENORRHEA.*

By WILLIAM S. GARDNER, M.D.,
BALTIMORE, MD.

It has been long recognized that painful menstruation is associated with, if not due to, some pathological condition, usually of such character that it can be readily recognized and often corrected. There are a limited number of cases where no gross lesion can be detected. And even when there occurs a gross lesion of the internal organs of generation, it does not necessarily follow that the dysmenorrhea is due to the lesion, or that the curing of such a lesion as is found will necessarily relieve the painful menses.

But from a careful clinical study of the lesions found associated with painful menstruation, we will learn more about the direct causal relations between the lesions and the pain, and consequently learn more about the relief of this trouble, which not only makes many a woman uncomfortable, but unfits her for duty during from ten per cent. to twenty-five per cent. of the active period of her life.

I have taken 120 cases that have complained of painful menstruation, and have attempted to classify them according to what appeared to be the most important lesion detected. Among the 120 were eight nulliparæ who, on account of an intact hymen, on the presumption of its presence, were not examined digitally; this leaves 112 who were examined.

One of the most striking points noted is the very large number of sterile women; forty-four, or a fraction less than forty per cent., belong to this class. Of those who had been pregnant, twelve, or over ten per cent., had never had a child at full term; fifteen

*Abstract of paper read before the Baltimore Obstetrical Society.

more, or thirteen per cent., had had a miscarriage since the last full term child was born, leaving less than thirty-seven per cent. of the total number whose last pregnancy had come to full term. Without further examination, these figures would indicate that, in a large proportion of patients suffering from dysmenorrhea, there were present lesions which also interfered with conception.

A detailed account of all these cases named would be very tedious, and I will give the list of lesions found, and then make some comments upon a part of them. It should be borne in mind that the lesion noted was not necessarily the only one present, but was the most marked, and presumably the one to which the pain was due. I say presumably, because we find these same lesions in patients who have no dysmenorrhea, but to go into the relations of all these lesions to dysmenorrhea, would lead us further than the limits of this paper would allow. Of the 112 patients examined, the following were found:

| | |
|---|---|
| Endometritis | 23 |
| Retroversions | 14 |
| Pyosalpinx | 17 |
| Anteflexions | 14 |
| Laceration of cervix and endometritis | 10 |
| Cervical stenosis | 8 |
| Constipation | 7 |
| Retroflexions | 4 |
| Enlarged ovaries | 4 |
| Fibroids | 2 |
| Prolapsed ovaries | 2 |
| Prolapsed uterus | |
| Lacerated cervix | 1 |
| Membranous dysmenorrhea | 1 |
| Nothing found | 5 |
| | 112 |

Of the twenty-three in whom endometritis was apparently the most marked lesion, nine had had their last pregnancies terminate in abortions; five are known to have had gonorrhea, and of the eight in whom nothing further than a cervical endometritis was noted, it is highly probable that a considerable number had had gonorrhea. Only one case that was probably a corporeal endometritis was noted.

Of the eighteen retrodisplacements, thirteen were retroversions in which no adhesions were detected; three, retroflexions, with no adhesions, and one retroflexion and one retroversion with adhesions. Two other retroversions, with pus tubes, are in the list of pyosalpinx cases.

Of the seventeen cases of pyosalpinx, nine were of gonorrheal origin, two probably puerperal, and the remaining six were due to an infection which could not be traced directly either to gonorrhea or puerperal infection.

Ten of the thirteen anteflexions had never been pregnant; the other three having been pregnant one or more times. At least one of the ten is known to have become pregnant after dilatation and gauze packing had been used. This patient had also had gonorrhea.

In three of the eight cases of stenosis of the cervix, the lesion was due either to a cicatrix forming after operation on the cervix, or after laceration.

The patient suffering from membranous dysmenorrhea made but one visit. She brought with her a complete cast of the interior of the uterus, and said that she had passed such a cast at each period since her last confinement, which had been five years previous to the visit.

From these cases it is seen that 100 out of 112 patients, suffering from painful menstruation, who were examined with a reasonable degree of care, were found to have some marked organic lesion of the internal generative organs.

The practical conclusion to be drawn is, that dysmenorrhea, being due in nearly all cases to some local trouble, the treatment for its relief must be directed toward relieving the local disease.

